# *Notes from the Field:* Large Cluster of Verona Integron-Encoded Metallo-Beta-Lactamase–Producing Carbapenem-Resistant *Pseudomonas aeruginosa* Isolates Colonizing Residents at a Skilled Nursing Facility — Chicago, Illinois, November 2016–March 2018

**DOI:** 10.15585/mmwr.mm6740a6

**Published:** 2018-10-12

**Authors:** Whitney J. Clegg, Massimo Pacilli, Sarah K. Kemble, Janna L. Kerins, Ahmed Hassaballa, Alexander J. Kallen, Maroya S. Walters, Alison Laufer Halpin, Richard A. Stanton, Sandra Boyd, Paige Gable, Jonathan Daniels, Michael Y. Lin, Mary K. Hayden, Karen Lolans, Deb P. Burdsall, Mary Alice Lavin, Stephanie R. Black

**Affiliations:** ^1^Chicago Department of Public Health; ^2^Epidemic Intelligence Service, CDC; ^3^Division of Healthcare Quality Promotion, National Center for Emerging and Zoonotic Infectious Diseases, CDC; ^4^Rush University Medical Center; Chicago, Illinois; ^5^APIC Consulting Services, Arlington, Virginia.

On November 1, 2016, a point prevalence survey conducted at a Chicago skilled nursing facility with ventilated residents (vSNF A) to understand the prevalence of carbapenemase-producing organisms in health care facilities in the Chicago region identified 20 patients with Verona integron-encoded metallo-beta-lactamase–producing carbapenem-resistant *Pseudomonas aeruginosa* (VIM-CRPA) colonization. To determine the extent of VIM-CRPA colonization at vSNF A and provide infection control recommendations, the Chicago Department of Public Health conducted an investigation.

The first VIM-CRPA outbreak reported in the United States occurred in a Chicago acute care hospital in 2003 (*1*). Other outbreaks have been described; however, none was associated with a single skilled nursing facility (*2*–*5*). Carbapenemase-producing CRPA are uncommon in the United States; a surveillance pilot for CRPA at five U.S. sites identified only two carbapenemase-producing CRPA among 129 isolates tested (CDC, unpublished data, 2017).

To determine whether ongoing transmission was occurring at vSNF A, the Chicago Department of Public Health conducted 10 additional point prevalence surveys during November 2016–March 2018. Rectal specimens were collected from all residents, and tracheostomy site specimens were collected from residents with tracheostomies. vSNF A is a licensed 312-bed facility; point prevalence surveys were conducted on a floor with standard skilled nursing (SN floor) and on a floor housing residents with a tracheostomy or who were mechanically ventilated (VT floor).

During November 2016–March 2018, collection of 903 screening swabs from 209 residents identified 38 residents with VIM-CRPA colonization. One additional colonized resident was identified by a rectal screening culture collected on admission to an acute care hospital. Among the 39 residents, four (10%) resided on the SN floor and 35 (90%) on the VT floor. Thirty (77%) had positive rectal swabs, four (10%) had positive tracheostomy swabs, and five (13%) had positive swabs from both sites.

Floor prevalence was calculated by dividing the number of VIM-CRPA–positive residents present on the day of the point prevalence survey by the total number of residents present. Prevalences ranged from 0% to 6% on the SN floor and 21% to 43% on the VT floor during November 2016–March 2018 (Table). Among the 18 additional residents with VIM-CRPA identified after the November 2016 point prevalence survey, 17 (94%) previously had screened negative at vSNF A, representing probable incident transmission events (Table). No additional residents with VIM-CRPA were identified during the last two consecutive point prevalence surveys on the SN floor and the last four on VT floors.

**TABLE:** Summary of Verona integron-encoded metallo-beta-lactamase–producing carbapenem-resistant *Pseudomonas aeruginosa* (VIM-CRPA) point prevalence surveys at vSNF A — Chicago, Illinois, November 2016–March 2018

Floor	Date of PPS	Total no. of residents on day of PPS	Newly identified VIM-CRPA	Newly positive, no previous screening	Newly positive, previously screened negative	Previously known VIM-CRPA	Total no. of residents positive on day of PPS	Prevalence, %
**SN**	11/1/16	72	4	4	0	0	4	6
	1/17/17	67	0	0	0	3	3	4
	7/10/17	71	0	0	0	0	0	0
**VT**	11/1/16	69	16	16	0	0	16	23
	1/9/17	66	2	0	2	12	14	21
	2/27/17	73	3	0	3	19	22	30
	5/10/17	73	4	1	3	19	23	32
	6/19/17	69	9	0	9	21	30	43
	7/5/17	74	0	0	0	30	30	41
	7/24/17	68	0	0	0	28	28	41
	11/20/17	67	0	0	0	28	28	42
	3/28/18	56	0	0	0	24	24	43
**Total**	**—**	**—**	**38**	**21**	**17**	**—**	**—**	**—**

**Abbreviations:** PPS = point prevalence survey; SN = standard skilled nursing floor; vSNF = skilled nursing facility with ventilated residents; VT = floor housing residents with tracheostomies or who were mechanically ventilated.

During November 2016–July 2017, point prevalence surveys were conducted at six other vSNFs and six long-term acute care hospitals in the Chicago region. Twelve additional VIM-CRPA positive patients were identified at five vSNFs and one long-term acute care hospital; facility prevalences ranged from 1% to 4%.

Whole genome sequencing performed on 26 isolates from five different facilities, including 19 from vSNF A, and two historical isolates from 2003 found that all contained the VIM-2 allelic variant, and 25 were multilocus sequence type (ST) 233 (others were ST 277, 708, and 875).* The ST233 isolates were identified across the five facilities and, using a core genome multilocus sequence typing analysis, clustered separately from epidemiologically unlinked ST233 isolates from CDC’s reference collection. In addition, clusters of highly related isolates (differences ranging from one to 10 single nucleotide polymorphisms) were consistent with transmission in vSNF A. These results suggest that a common strain of VIM-CRPA has had a longstanding presence in this region, with recent transmission in vSNF A.

The Chicago Department of Public Health provided ongoing on-site assessments to monitor infection control practices. Improvements were made in hand hygiene and isolation precautions compliance, resident cohorting, bathing practices, and environmental cleaning. The facility also stopped rinsing respiratory equipment with tap water in sinks in residents’ rooms.

This is the largest health care–associated cluster of VIM-CRPA isolates colonizing residents reported in the United States to date. Although centered in one vSNF, this investigation highlights the interconnectedness of health care facilities through patient sharing and how prolonged, undetected transmission can result in spread through a region. Application of CDC’s multidrug-resistant organisms containment guidance (*6*), including comprehensive on-site assistance and colonization screening, limited transmission at the index facility despite continued high prevalence. Improved availability of carbapenem resistance mechanism testing and screening tests are critical for early identification of and response to similar clusters. These resources are now available through CDC’s Antibiotic Resistance Laboratory Network.

## Footnotes

* Raw sequencing reads were placed under BioProject ID PRJNA474674.
